# Enhancing Cutting Oil Efficiency with Nanoparticle Additives: A Gaussian Process Regression Approach to Viscosity and Cost Optimization

**DOI:** 10.3390/nano15131008

**Published:** 2025-06-30

**Authors:** Beytullah Erdoğan, İrfan Kılıç, Abdulsamed Güneş, Orhan Yaman, Ayşegül Çakır Şencan

**Affiliations:** 1 Department of Mechanical Engineering, Engineering Faculty, Zonguldak Bülent Ecevit University, Zonguldak 67100, Turkey; aysegulcakir@beun.edu.tr; 2 Department of Software Engineering, Engineering Faculty, Fırat University, Elazig 23119, Turkey; irfankilic@firat.edu.tr; 3 Department of Electrical and Energy, Elazığ Organized Industrial Zone Vocational School, Fırat University, Elazig 23119, Turkey; agunes@firat.edu.tr; 4 Department of Digital Forensic Engineering, Technology Faculty, Fırat University, Elazig 23119, Turkey; orhanyaman@firat.edu.tr

**Keywords:** cutting fluid, Gaussian process regression (GPR), nanofluid, dynamic viscosity, fitness function, cost analysis

## Abstract

Nanoparticle additives are used to increase the cooling efficiency of cutting fluids in machining. In this study, changing dynamic viscosity values depending on the addition of nanoparticles to cutting oils was investigated. Mono nanofluids were prepared by adding hBN (hexagonal boron nitride), ZnO, MWCNT (multi-walled carbon nanotube), TiO_2_, and Al_2_O_3_ as nanoparticles, hybrid nanofluids were prepared by using two types of nanoparticles (ZnO + MWCNT, hBN + MWCNT etc.), and ternary nanofluids were prepared by using three types of nanoparticles. GPR (Gaussian process regression) was used to estimate unmeasured dynamic viscosity values using the dynamic viscosity values measured for different temperatures. Dynamic viscosity results are a precise determination (R^2^ = 1). An augmented dataset was obtained by adding the dynamic viscosity values estimated with high accuracy. A fitness function based on dynamic viscosity and nanoparticle unit costs was proposed for the cost analysis. With the help of the proposed fitness function, it was observed that the best performing nanoparticles were the ZnO and ZnO hybrid mixtures according to different dynamic viscosity and cost effects. The study showed that the most suitable nanofluid selection focused on performance and cost could be made without performing experiments under various operating conditions by increasing the limited experimental measurements with strong GPR estimates and using the proposed fitness function.

## 1. Introduction

In machining methods, some of the cutting energy is converted into heat during the process, causing temperatures in the cutting zone to increase. In addition, friction creates even higher temperatures in the cutting zone. In addition to the wear of the cutting tools, this situation causes the surface quality of the processed material to deteriorate, and more power to be needed in the cutting process. Traditionally, coolants, especially cutting oils, are used to eliminate this problem. However, in order to reduce the high temperature and pressure effects caused by 1processing technologies, innovative solutions are needed, i.e., using nanotechnology and increasing the thermal conductivity and dynamic viscosity values of the fluid by adding nanoparticles to the basic fluids and cutting oils. Öndin et al. made a comparison with the MQL (minimum quantity lubrication) method in the machining of pH 13–8 Mo stainless steel using MWCNT nanoparticles in the cutting fluid. It was determined that the cutting fluid with MWCNT added reduced surface roughness by approximately 12%, cutting temperature by 38%, and tool wear by 69%. These results show that the use of nanoparticles provides superior properties compared to the MQL method [1].

Lee et al. showed that the thermal conductivity of Al_2_O_3_–water nanofluids increases almost linearly with concentration, but the viscosity decreases with temperature and shows a nonlinear relationship with concentration, which is different from what was expected [2].

Bag et al. investigated the use of nanofluid cooling technique in machining AISI 4340 hardened steel and its potential to increase the thermal conductivity of cutting fluids. It is emphasized that nanoparticles with high heat dissipation capacity, especially Al_2_O_3_, can increase machining performance with their cooling and lubrication properties [3]. Sharma et al. investigated the effect of MoS_2_ and Al_2_O_3_ nanoparticles on the turning of AISI 304 steel. In a study conducted at the rates of 0.25–1.25%, Al_2_O_3_ increased the viscosity by 152%, while the Al_2_O_3_–MoS_2_ mixture increased the viscosity by 71% [4]. Yadav et al. investigated the cutting forces, especially in turning operations, by using single and hybrid nanoparticles in metal cutting processes. It was stated that the use of hybrid nanofluid at concentrations of 0.25–0.5–0.75% had 7.6% less cutting force compared to single nanofluid [5].

Ravi and his colleagues stated that the use of nanofluids in metalworking processes provides significant improvements in processing parameters, and = improvements in such properties as viscosity and thermal conductivity play an especially important role [6]. In their experimental studies, Gajrani and his colleagues used calcium fluoride (CaF_2_) and molibdenum disulfide (MoS_2_) nanoparticles in addition to standard mineral oil and mixed them at different volumetric concentrations, then performed cooling processes in metalworking with a hybrid nanofluid called HN-GCF (hybrid nano-green cutting fluid). As a result of the study, it was stated that HN-GCF nanofluid showed better performance, with 37% more surface quality than other cutting fluids [7]. Selvarajoo et al. measured the thermophysical properties, such as viscosity and thermal conductivity, of hybrid nanofluid mixtures made using Al_2_O_3_ and graphene oxide at different volumetric concentrations. Experimentally, an analytical regression method was used to estimate the viscosity and thermal conductivity values in other properties. It was reported that the thermal conductivity value of the hybrid nanofluid was 4.30% higher for Al_2_O_3_ and 4.34% higher for GO (graphene oxide) at 1% volumetric concentration [8]. Singh et al. reported that the thermal conductivity and viscosity of the hybrid nanofluid they created using aluminum and graphene nanoparticles as cutting fluid in metalworking increased as the volumetric concentration increased, while the surface roughness decreased by 20.28%, the cutting force decreased by 9.94%, and the thrust force decreased by 17.38% [9].

The effects of nanocutting fluids prepared with MWCNT and Al_2_O_3_ nanoparticles on surface roughness, environmental impact, and power consumption were investigated. Increased viscosity improved heat transfer while increasing power consumption. Predicting these effects with artificial intelligence will be beneficial not only in terms of numerical data but also in terms of process regulation [10]. Hirudayanathan et al. studied the effects of size, shape, material, and distribution of nanoparticles on nanofluid viscosity and processing properties. Ceramic-based nanofluids are more stable, while metallic and carbon-based ones show more sedimentation and poor performance. Viscosity increases as particle size decreases; however, evaluating this relationship without artificial intelligence may lead to erroneous results. In addition, the strong interaction between the base fluid and nanoparticles causes agglomeration, negatively affecting the surface quality [11].

As a result of experimental studies conducted by Manikanta et al., it was determined that antifriction and cooling rate increased with the increase in the volumetric concentration ratio of nanoparticles added to base fluids [12]. In the study conducted by Duc et al., it was determined that the minimum amount of lubrication with Al_2_O_3_ nanofluid in cutting fluids used in drilling of Hardox 500 steels showed higher viscosity, better heat transfer, better surface quality, and lower cutting force compared to MQL with pure water [13]. Hybrid nanofluid formed by using Al_2_O_3_ and TiO_2_ nanoparticles together was used in cutting fluids in high-speed machines by Arifuddin et al. In the MQL study, the effects of surface roughness, cutting temperature, and tool heating were evaluated. Al_2_O_3_–TiO_2_ nanofluid used at 4% volumetric concentration gave the highest surface roughness reduction rate due to the increase in viscosity [14]. Singh et al. used MoS_2_, TiO_2_, Al_2_O_3_, and SiO_2_ nanofluids at various concentrations, such as 0.2% and 2%, in such processes as turning, milling, drilling, and grinding, and positive results were reported [15]. Prabhu et al. prepared a SAE20W40 based nanofluid with 10–20 nm MWCNT nanoparticles at a 0.02% volume fraction and investigated the effect of this fluid on surface roughness. An ANN (artificial neural network) method was used in the analysis for all variables, and low prediction errors between 0.88–9.23% were obtained with the developed fuzzy logic model [16]. A ternary hybrid nanofluid containing TiO_2_, ZnO, and Fe_2_O_3_ nanoparticles was prepared by Vignesh et al. using coconut oil as the base fluid and then used as cutting fluid. Improved results were obtained in cutting forces, tool wear, and surface roughness with the increase in viscosity in lubrication and cooling processes [17]. Elshazly et al. investigated the thermal performances of MWCNT, Al_2_O_3_, and 50% mixed hybrid MWCNT/Al_2_O_3_ nanofluids and found that the hybrid nanofluid provided a 18–29% increase in efficiency [18].

Thermophysical properties were investigated by Azharuddin et al. using AgNO_3_/graphene nanoparticles. In this study using a hybrid nanofluid, thermal conductivity, viscosity, and specific heat properties were investigated. As a result of the studies, thermal conductivity increased by 8.21%, 15.37%, and 23.59% at various concentration ratios, respectively [19]. Riyadi et al. discussed the advantageous situations created by the use of machine learning methods in the use of nanofluids. In this context, it was stated that such methods made significant contributions to the increase and prediction of thermal efficiency in heat transfer processes, especially in the energy field. In addition, a decrease in energy consumption and total cost was observed with the use of this combination in heat exchangers [20]. In their study using ternary nanofluid (CoFe_2_O_3_ + Cu + Al_2_O_3_/H_2_O), hybrid nanofluid (CoFe_2_O_3_ + Al_2_O_3_/H_2_O), and single-component nanofluid (CoFe_2_O_3_/Al_2_O_3_/H_2_O), they reported that the increase in radiation and convective heat transfer processes increased the thermal efficiency in ternary nanofluid compared to other fluid types [21]. In the study conducted by Humaira Yasmin et al., the fluid behavior of ternary hybrid nanofluids on the tension plate was investigated according to the Reynolds viscosity model depending on the temperature [22]. In the study conducted by Ruihao Zhang et al., the effect of single (Ag, Cu, Au, and Fe) and hybrid (Au–Ar) nanoparticles on viscosity was investigated [23]. When the literature is examined, it is understood that the use of nanofluid as a cutting fluid in machining provides high efficiency in terms of material surface quality. In addition to sunflower oil, rapeseed oil and olive oil have also been used in these processes [24]. While the lubricating feature of the cutting fluid is needed more in the case of high cutting depth in metalworking, the cooling effect of the cutting fluid is needed more in high-speed cutting operations. There is no comprehensive study that will provide the most appropriate estimate and suggestion regarding which nanofluid to use at which concentration in these processes by providing optimum viscosity values under various conditions or the most important criterion, namely the nanofluid cost. In existing studies, the extremely high prices of nanoparticles are not mentioned in the methods used. In studies carried out in this way, very high prices emerge and the possibility of putting them into practice is almost impossible. In the study conducted for these needs, it was aimed to determine the conditions that provide the highest processing performance regardless of the cost in the preparation of the most efficient nanofluid in metalworking processes and also to provide minimum cost cooling and lubrication. In addition, the study focused on determining the most accurate cutting oil feature for sustainable and environmentally friendly production by providing both criteria at certain rates. For this purpose, an environmentally friendly vegetable oil (sunflower oil) was used as the base fluid. Various nanofluids were prepared by incorporating six different nanoparticles in mono, hybrid, and triple hybrid forms into the plant base fluid. The dynamic viscosity properties affecting the wetting and lubrication ability of the prepared nanofluids were measured at four different temperature conditions and intermediate values were estimated by numerical/analytical methods. In this context, it is thought that the study conducted will shed light on the relevant researchers by providing the opportunity to determine the properties of nanofluids that will be used as cutting fluids that will provide high performance and reduce costs in the processing area.

### 1.1. Motivation and Contributions

The use of nanofluids in cutting fluids has yielded quite successful results in the literature. However, in different machining processes, such as turning, milling, drilling, honing, planing, and grinding, the most suitable nanofluid type, concentration, and viscosity value have not yet been clearly determined according to the process type, process parameters, and application conditions. A reliable and comprehensive estimation method that evaluates criteria, such as cost, cooling performance, and machinability, together is lacking.

This study aims to develop a system that provides both maximum cooling performance and minimum cost alternatives for each process, and suggests these two criteria in a balanced manner when necessary. Although nanoparticle-added fluids (single, hybrid, ternary) significantly increase dynamic viscosity, measuring this viscosity in different temperature ranges in a laboratory environment is quite costly and time-consuming. Therefore, existing datasets are limited and temperature ranges are incomplete. In addition, there is no comprehensive experimentally validated method for selecting the ideal nanofluid.

As a result of this study, the following contributions were presented:

• Bringing two measured and augmented datasets to the literature for different fluids.
• Obtaining a reliable augmented dataset from measured values using Gaussian process regression.
• Proposing a fitness function for the analysis of dynamic viscosity and nanofluid costs.
• The most suitable nanofluid selection can be made with the proposed fitness function.
• It provides scientific evidence for researchers on which nanofluid can be processed with high efficiency for which machining process, at which volumetric concentration, and at the most affordable cost.

### 1.2. Paper Organization

This paper is organized as follows. In the introduction, after explaining the importance of increasing the dynamic viscosity, previous studies are mentioned. Then, the main challenges are presented and the main contributions obtained as a result of the study are presented. In Section 2, after the preparation of nanofluids and the dynamic viscosity measurement process are explained, the obtained datasets are shown. It is shown how these datasets are augmented with the help of GPR. In Section 3, mono, hybrid, and ternary nanofluids are analyzed with augmented data. A fitness function that takes into account the viscosity values of the augmented data and the unit costs of nanofluids is proposed and analyzed and discussed accordingly. In Section 4, the general conclusions and future studies are mentioned.

## 2. Materials and Methods

### 2.1. Nanofluid Preparation

In the study, environmentally friendly and human health-friendly sunflower oil was specifically selected as the base fluid. Vegetable oils have advantages in terms of their technical properties and in terms of preserving the oil form at the tool–chip interfaces under heavy conditions in machining. In addition, sunflower oils commercially produced specifically for machining by adding some additives can give better results in terms of machining performance [25]. In this study, sunflower oils specially produced for machining were used. Their technical properties are as in Table 1.

To convert the base fluid into nanofluid, 5 different nanoparticles were added in mono, hybrid, and ternary hybrid forms. These nanoparticles were hBN, ZnO, MWCNT, TiO_2_, and Al_2_O_3_. In the selection of these nanofluids, nanoparticles with high viscosity and thermal conductivity properties were selected from the literature. The properties of the nanoparticles used are given in Table 2.

**Figure 1:**
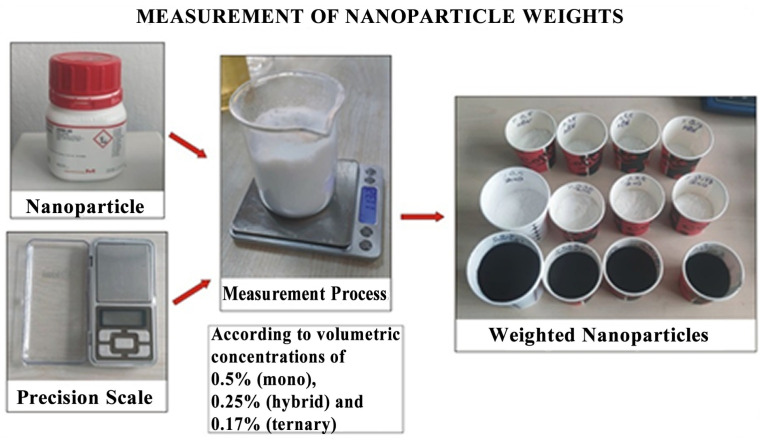
Measurement of particle weights according to masses corresponding to volumetric concentrations.

In the study, a total of 13 different nanofluids (5 mono, 6 hybrid, and 2 ternary hybrid) were prepared using the 2-stage method. In mono-fluids where all nanoparticles in Table 2 were used separately as a single type, the particle to fluid volumetric concentration ratio was 0.5%. In hybrid nanofluids where two types of nanofluids were used, each particle was added at an equal concentration (0.25%) so that the total particle to fluid ratio would again be 0.5%. The nanofluids prepared as hybrids were ZnO + MWCNT, hBN + MWCNT, hBN + ZnO, hBN + TiO_2_, hBN + Al_2_O_3_, and TiO_2_ + Al_2_O_3_. In ternary hybrid nanofluids where three types of nanoparticles were used, the volumetric ratio of total particles to the base fluid was 0.5%m and they were added at equal concentrations (0.17%). The nanofluids prepared as ternary hybrid mixtures were hBN + ZnO + MWCNT and hBN + TiO_2_ + Al_2_O_3_. While preparing these nanofluids to be used as cutting fluids, the amounts of nanoparticles were first determined with a precision balance (ANDGX—600, Cihazlab Inc., Ankara, Turkey, maximum mass: 610 g, deviation: 0.001 g). Since the volumetric concentration of a nanoparticle type should be 0.5%, 0.25%, and 0.17%, respectively, when it is included in mono, hybrid, and ternary hybrid nanofluids, the volumetric data for each were converted to mass data using the formulas in Table 3 [26]. The mass data of the nanoparticle to be added, determined in this way, were measured on a precision scale and placed in separate containers (Figure 1).

**Figure 2:**
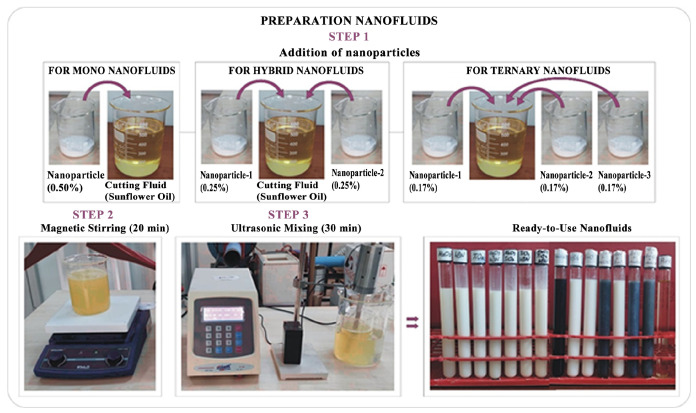
Steps applied in the preparation of nanofluids.

For the preparation of nanofluids, nanoparticles and base fluid (vegetable oil) weighed on a precision scale were mixed with a magnetic stirrer for 15–20 min, and then, in order to obtain a more homogeneous fluid, the solution was mixed with an ultrasonic homogenizer (brand/model: Optical Ivy System CY—500, TETRA Technological Systems Inc., İstanbul, Turkey, power: 500 W, frequency: 20 kHz, probe diameter/length: Ø5, 6/60 mm) for at least 30 min and made ready for use. Cutting fluids were prepared so that the nanofluid required for each experiment would be 0.5 L. The steps applied in the preparation of cutting fluids are given schematically in Figure 2.

### 2.2. Viscosity Measurement

**Figure 3:**
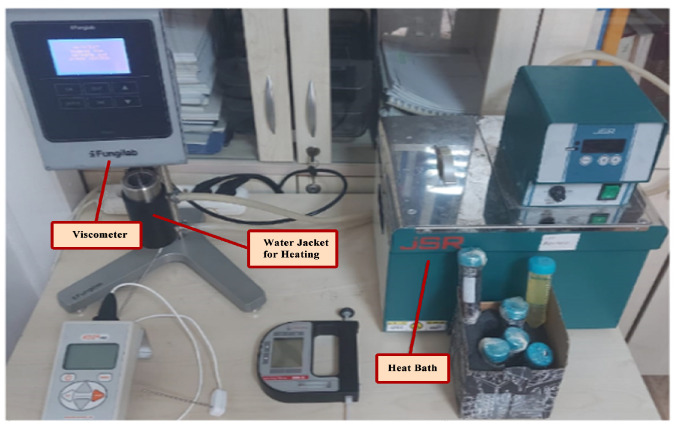
Dynamic viscosity measurement process.

Dynamic viscosity measurements of nanofluids were made with a Fungilab Smart L device (±2%) with a measurement range of 0–2000 mPa·s, as given in Figure 3. Experiments were performed at standard settings of the device and a constant shear rate of 10 rpm. Measurements were made at constant temperature (approximately 30 °C) and using the same geometry for 12 different nanofluids, so that all samples were evaluated under comparable conditions. An LCP (low viscosity adapter) was used in viscosity measurements of nanofluids prepared with a minimum of 20 mL. Dynamic viscosity (μ) values were measured at the desired temperatures by heat bath in the water jacket in the viscosity measuring device and repeated at least 6 times.

### 2.3. Dataset

In the laboratory setup and measurement system given in Figure 3, 30 different dynamic viscosity values were measured for each fluid in the temperature range of 30–70 °C for single, hybrid, and ternary nanofluid mixtures. Table 4 shows the measured viscosity values of 5 different nanofluids prepared using pure oil and a single nanoparticle. Table 5 shows the measured viscosity values of 6 different hybrid nanofluids using 2 different nanoparticles. Table 6 shows the measured viscosity values of 2 different ternary nanofluids prepared using 3 different nanoparticles. When the measured values in Table 4, Table 5 and Table 6 are examined, it is seen that the temperature values are not in an ordered range (e.g., 30.00, 30.10, 30.20, 30.30…). In addition, the total number of measurements, 390, is very few. It is quite difficult to measure all the values in the range in an ordered manner according to temperature in terms of time and cost. Instead, it is necessary to estimate the measurements in a certain range with a high-accuracy method using the available measurements. In this way, it will be more consistent to analyze the data with sufficiently increased measurements.

### 2.4. Our Method

**Figure 4:**
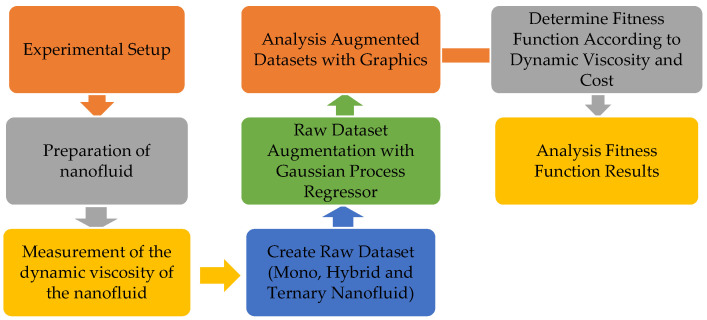
Framework of our proposed method.

After obtaining the dataset, the dataset measurements were augmented using GPR, which is a powerful regression method for estimating the values in between. The general framework of the applied method is given in Figure 4. In this context, when the general framework of the method we propose is examined, the experimental data obtained by making 30 different measurements for each single, hybrid, and ternary nanofluid with the experimental setup set up in the laboratory environment are obtained by augmented datasets for 3 datasets with the help of GPR.

These datasets were examined with graphics for each nanofluid type according to temperature and viscosity values. A fitness function was determined that takes into account the nanofluid costs (C) and dynamic viscosity (DV) values. The fitness values were obtained by changing the weights of these variables in the range of [0–1] depending on the C and DV variables. The fitness values obtained were analyzed for each nanofluid.

### 2.5. Gaussian Process Regression (GPR)

GPR is one of the powerful methods used in estimation and regression problems in machine learning and statistics. This method works with a nonparametric approach that provides flexible modeling by using the correlation structure between data points. GPR has the capacity to make probabilistic predictions for future values over a model trained with sequential data [27,28,29]. The basic equations of GPR are presented below.

Gaussian processes (GPs) can be thought of as a family of probability distributions. In a system where any function *f*(*x*) can be modeled, a Gaussian distribution is predicted at all points of that function. A Gaussian process operates as a “function distribution”; that is, it provides a priori information about the function we are modeling. In the GPR model, the relationship between any two data points is determined by the covariance function *k*(*x*,*x*′). If a Gaussian process is to be applied to the function *f*(*x*), it can be expressed as in the following Equation (1):

(1) $$f(x)∼GP(m(x),k(x,x^{\prime }))$$

where

m(x)=E[f(x)] is the average function is taken as 0 in most cases.
*k* (*x*, *x*′) = *Cov* (*f*(*x*), *f*(*x*′)) is the covariance function or kernel function.

The covariance function (kernel function) is the most effective measure for describing the relationship between two data points. The radial basis function (RBF) kernel function is expressed as the following Equation (2):

(2) $$k(x,x^{\prime })=\sigma_{f}^{2}\exp\left(-\frac{{\left\|x-x^{\prime }\right\|}^{2}}{{2l}^{2}}\right)$$

where *σ_f_* is a hyperparameter that determines the scale of the function’s output, and *l* is the hyperparameter that determines the smoothness of the function (the length of the spatial correlation).

In this study, single-task GPR was used as the prediction model. In the model, a Matérn 5/2 function was preferred as the kernel (covariance) function that defines the relationship between data points. Matern kernel functions are powerful kernels widely used in regression problems due to the flexibility and different smoothness levels they provide. Values, such as length scale (*ℓ*), signal variance $\left(\sigma_{f}^{2}\right)$ and noise level $\left(\sigma_{n}^{2}\right)$, which are the hyperparameters of the GPR model, were not determined manually, but instead were automatically optimized to maximize the marginal probability (log-evidence) function of the training data. This approach reduces the risk of overfitting by adjusting the complexity of the model appropriately to the data. In addition, the data were standardized before model training (scaled to have a mean of 0 and unit variance for the inputs and target variable). This preprocessing step facilitated numerical parameter optimization, allowing the GPR model to learn more stably and quickly. The optimization algorithm used during the training of the GPR model worked in an integrated manner with the default methods of the GPR library and found the numerically best values of the hyperparameters. As a result, with the selected kernel type and automatic hyperparameter tuning, the GPR model was configured to perform flexible learning appropriate to the structure of our dataset.

*Gaussian Process Regression (GPR)*

The aim of GPR is to model the dynamic viscosity output $Y={\left\{y\right\}}_{i=1}^{n}$ corresponding to a certain temperature value $X={\left\{x\right\}}_{i=1}^{n}$. In this case, we assume that the function f(x) is an unknown model and express our observations as corrupted by noise (*ϵ*), as in the following Equation (3):

(3) $$y_{i}=f(x_{i})+\epsilon_{i}$$

where Gaussian noise with a zero mean $\epsilon_{i}=\sim N\;(0,\;\sigma_{n}^{2})$ and range $\sigma_{n}^{2}$ is added. After establishing this model, the following steps are followed when making a prediction for a new data point *x*_∗_.

*Establishment of Joint Distribution*

In GPR, observations and predictions together form a multivariate Gaussian distribution. With the outputs of the training data y and the output of the new data point to be predicted *f*_∗_, the joint distribution can be written as in the following Equation (4):

(4) yf∗ ~ N 0,  KX,X+σn2IK(X,x∗)K(x∗,X)K(x∗,x∗)

where *K* (*X*, *X*) is the covariance matrix between training data points, *K* (*X*, *x*_∗_) is the covariance vector between training and test data points, and *K* (*x*_∗_, *x*_∗_) is the covariance (a scalar) expression for the test data.

*Obtaining The Posterior Distribution*

Starting from the joint distribution, the conditional distribution is used to calculate the output for the test point *x*_∗_. Due to the nature of Gaussian processes, the conditional distribution will also be a Gaussian distribution and is calculated as follows in Equation (5):

(5) $$f_{*}\;|\;X,y,x_{*}∼N\;({f^{-}}_{*},Var(f_{*}))$$

where the estimated value and variance are expressed as follows, respectively, in Equations (6) and (7):

(6) $${f^{-}}_{*}=K\left(x_{*},X\right){\left[K\left(X,X\right)+\sigma_{n}^{2}I\right]}^{-1}y$$

(7) $$Var\left(f_{*}\right)=K\left(x_{*},x_{*}\right)-K\left(x_{*},X\right){\left[K\left(X,X\right)+\sigma_{n}^{2}I\right]}^{-1}K(X,x_{*})$$

These equations provide the expected value (*f*^−^_∗_ = *y*′) of the estimate and the measure of uncertainty [variance (*Var*(*f*_∗_)]. For all three datasets (mono, hybrid, ternary), 400 values were estimated according to the calculations given in Equations (1)–(7). In order to test the robustness of the obtained values to the measured values, mean square error (MSE), root mean square error (RMSE), mean absolute error (MAE), and R-square (R^2^) values were calculated as given in the following Equations (8)–(11) (Table 7):

(8) $$MSE=\frac{1}{n}\sum_{1=1}^{n}{\left(y_{i}-y^{\prime }\right)}^{2}$$

(9) $$RMSE=\sqrt[{2}]{\frac{1}{n}\sum_{1=1}^{n}{\left(y_{i}-y^{\prime }\right)}^{2}}$$

(10) $$R^{2}=1-\frac{\sum_{i=1}^{n}{\left(y_{i}-y^{\prime }\right)}^{2}}{\sum_{1=1}^{n}{\left(y_{i}-z^{\prime }\right)}^{2}}$$

(11) $$MAE=\frac{\sum_{i=1}^{n}\left|y_{i}-{y^{\prime }}_{i}\right|}{n}=\frac{\sum_{i=1}^{n}\left|e_{i}\right|}{n}$$

where *n* is the number of samples in the data, *y_i_* is the actual value (target variable), *y*′ is the average value estimated by GPR, *z*′ is the mean of the target variable, and *e_i_* is the absolute error at i rank.

## 3. Experimental Results and Discussion

**Figure 5:**
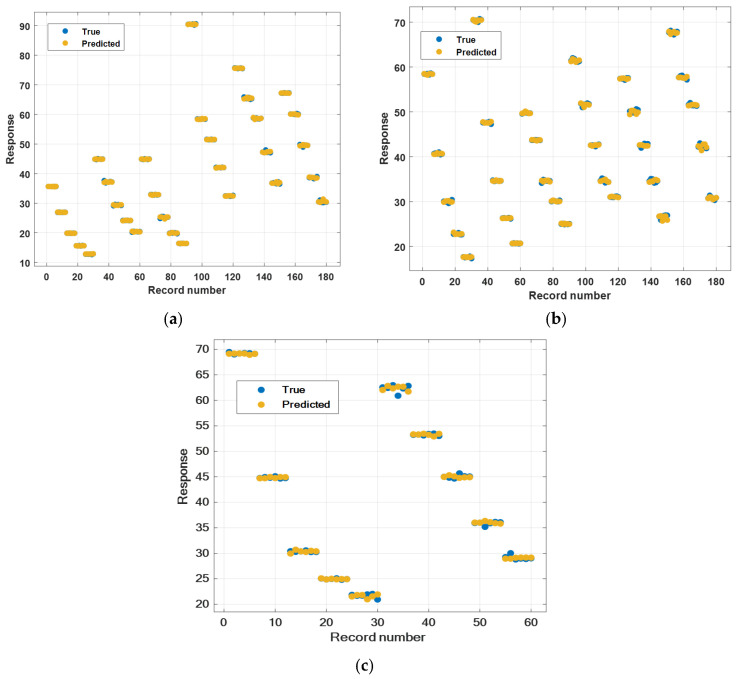
Response–record number graph of GPR model. (**a**) Mono. (**b**) Hybrid. (**c**) Ternary.

Figure 5 shows the graphs of the measured and GPR-predicted dynamic viscosity values of three (single, hybrid, and ternary) nanofluid mixtures. The measured values are shown in blue and the GPR predicted values are shown in yellow. As can be seen in the graphs, the predicted values overlap with the measured values. Table 7 shows the MSE, RMSE, MAE, and R^2^ values obtained using GPR, neural network, support vector machine (SVM), and regression trees and the training times spent calculating these values. It will be seen that MSE, RMSE, MAE, and R^2^ metrics for nanofluid mixtures (single, hybrid, ternary) were obtained with GPR. The best result in terms of training time for single nanofluids was obtained with SVM. Regression trees gave the best training time for hybrid and ternary nanofluids. The training time for GPR is an acceptable time considering the number of predicted values. The strong estimates seen in the graph in Figure 5 are also confirmed by the value of R^2^ = 1 (for GPR).

In order to verify that the GPR model does not exhibit overfitting and to confirm its generalization ability, a performance evaluation was performed using the 10-fold cross-validation method. In this method, the dataset was randomly divided into 10 subsets, the model was trained on 9 subsets in each iteration, and the remaining 1 subset was used for testing; this process was repeated for all subsets. Performance metrics, such as MSE, RMSE, MAE, and R^2^ (coefficient of determination), were calculated as a result of each fold. The results showed that the error metrics were quite stable among all folds. For example, the RMSE and MAE values calculated for each fold of the GPR model showed small deviations around the mean, while the R^2^ values were consistently high across all folds (around R^2^ ≈ 1.0 in each fold). The fact that the performance scores are similar in all validation sets proves that the model does not overfit the data, but on the contrary, it can make successful predictions on new data. In particular, if the model was prone to overfitting, it would be expected to see significant fluctuations in performance scores between different folds. However, no such fluctuations were observed in the cross-validation results for the GPR model, indicating that the overall validity of the model is high. In addition, the summary results of this cross-validation evaluation are presented in a graph or table. This visual presentation shows the distribution of errors and R^2^ values between the predicted and actual values for each fold, clearly demonstrating that the GPR model can achieve high prediction performance even at unknown temperature values. In this way, it has been confirmed that the proposed GPR model gives reliable results not only in the training data but also in new temperature ranges that it has not seen.

**Figure 6:**
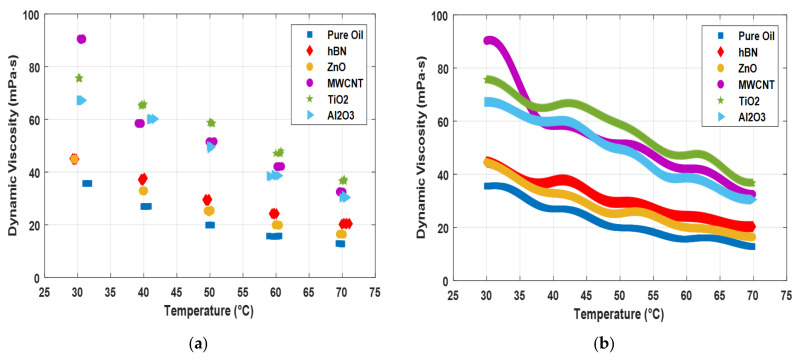
Temperature and dynamic viscosity values of nanofluids. (**a**) Raw dataset; (**b**) results obtained with the GPR model.

Figure 6 shows the temperature–dynamic viscosity graph of the augmented dataset to which the experimental data of single nanofluids and the values obtained with GPR are added. The graph shows the dynamic viscosity change depending on the temperature increase. As can be seen from the graph, a dashed appearance was obtained because there were missing data in the experimental data. With the augmented data, the change in viscosity of the nanoflows depending on the temperature can be clearly observed. It is seen that single nanofluids perform better than pure oil in terms of viscosity. While MWCNT performs better at low temperatures, it is seen that TiO_2_ performs better as the temperature increases. It is seen that the highest dynamic viscosity value of single nanofluids at high temperatures is TiO_2_ (~38 mPa·s).

**Figure 7:**
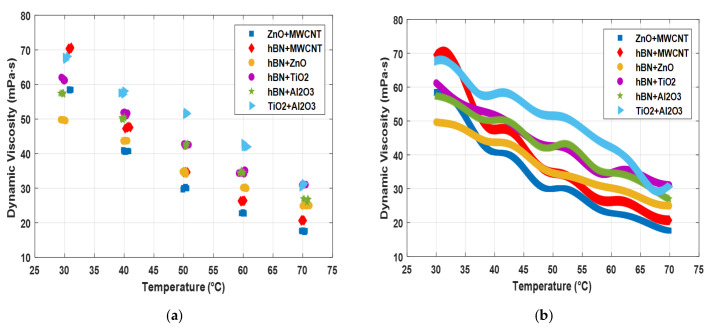
Temperature and dynamic viscosity values of hybrid fluids. (**a**) Raw dataset; (**b**) results obtained with the GPR model.

Figure 7 shows the temperature–dynamic viscosity graph of the raw dataset of hybrid nanofluids and the augmented dataset to which the values obtained with GPR are added. The graph shows the dynamic viscosity change depending on the temperature increase. As can be seen from the graph, a dashed appearance was obtained because there were missing data in the raw dataset. The change in the viscosity of hybrid nanofluids with temperature can be clearly observed with the augmented data. While the hBN + MWCNT hybrid mixture and the TiO_2_ + Al_2_O_3_ hybrid mixture show better performance at low temperatures, it is seen that the TiO_2_ + Al_2_O_3_ and hBN + TiO_2_ hybrid mixtures show better performance as the temperature increases. It is observed that the highest viscosity value at the highest temperature (~70 °C) is the hBN + TiO_2_ hybrid mixture (~31.5 mPa·s).

**Figure 8:**
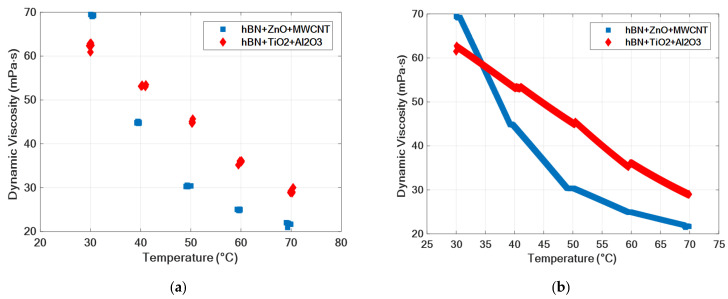
Temperature and dynamic viscosity values of hybrid fluids. (**a**) Raw dataset; (**b**) results obtained with the GPR model.

Figure 8 shows the temperature–dynamic viscosity graph of the raw dataset of ternary nanofluids and the augmented dataset to which the values obtained with GPR were added. The graph shows the dynamic viscosity change depending on the temperature increase. As can be seen from the graph, a dashed appearance was obtained because there were missing data in the raw dataset. The change in the viscosity of hybrid nanofluids with temperature can be clearly observed with the augmented data. While the hBN + ZnO + MWCNT ternary mixture shows better performance at low temperatures, it is seen that the hBN + TiO_2_ + Al_2_O_3_ ternary mixture shows better performance as the temperature increases.

Table 8 shows the unit prices of single nanofluid, hybrid, and ternary mixtures per 100 g. In choosing which nanofluid or mixture, costs should be considered as well as dynamic viscosity. Accordingly, a fitness function given in Equation (12) is proposed. The aim of this study is to determine the maximum viscosity and the minimum price. The proposed fitness function is given in Equation (12).

According to Equation (12), V represents the viscosity of the mixture and F represents the cost of the mixture. The *V_max_* and *F_max_* values are the highest viscosity and price values observed in the existing mixtures. These values are normalized to be between 0 and 1. The μ and EUR values are the coefficients that determine the relative importance of viscosity and price. The μ and EUR weights can take values in the range of [0, 1]. According to Equation (12), as the viscosity value increases, the fitness value will increase, and as the price increases, the fitness value will decrease. High fitness value means high viscosity and low-cost mixtures. Equation (12) is as follows:

(12) $$Fitness=\mu *\;\frac{V}{Vmax}+EUR*\;\frac{1}{\left(\frac{F}{Fmax}\right)}$$

V = Viscosity value at i rank, Vmax = Highest viscosity value, Fmax = Highest unit cost.

**Figure 9:**
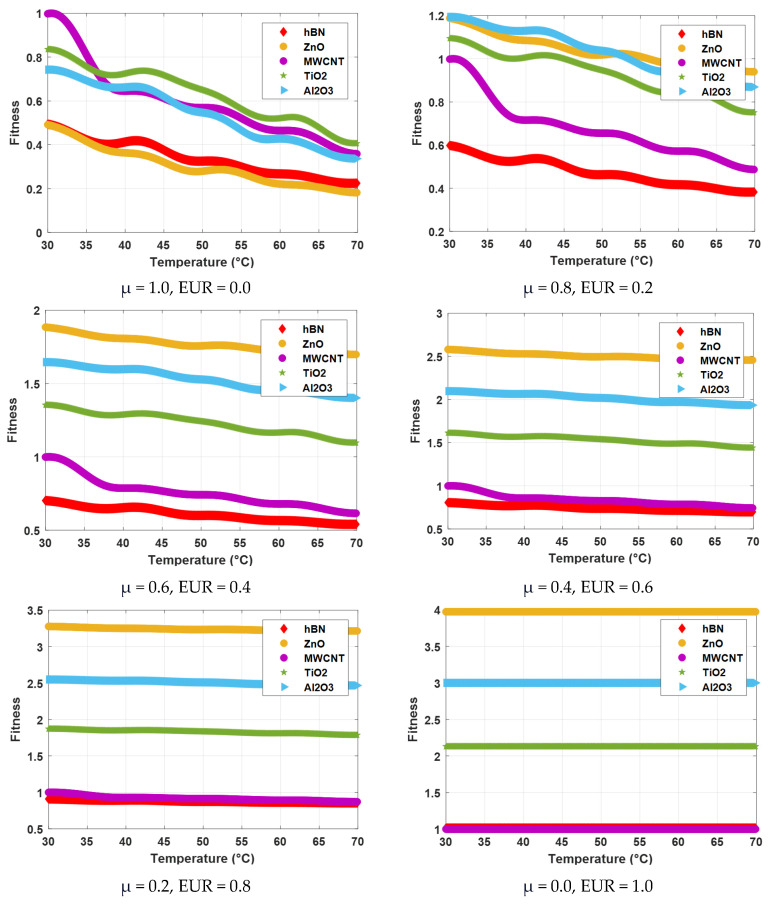
The fitness function graph obtained using viscosity and price parameters of nanofluids.

Figure 9 shows the graphs of fitness values of single nanofluids calculated depending on temperature increase according to different viscosity weights (μ) and different price weights (EUR). In the case where viscosity is 100% (μ = 1, EUR = 0), MWCNT has the highest fitness value at the beginning, while TiO_2_ gives the highest fitness value at high temperatures. In the case where viscosity is 80% and the price is 20% (μ = 0.8, EUR = 0.2), the Al_2_O_3_ fitness value is high at low temperatures, while the ZnO fitness value is the highest at high temperatures. The ZnO fitness value is the highest at all temperatures where viscosity is 60% and the price is 40% (μ = 0.6, EUR = 0.4). The ZnO fitness value is again the highest at all temperatures where viscosity is 40% and the price is 60% (μ = 0.4, EUR = 0.6). It is seen that the ZnO fitness value stands out significantly compared to other fluids at all temperatures where the viscosity is 20% and the price is 80% (μ = 0.2, EUR = 0.8) and when the viscosity is 0% and the price is 100% (μ = 0, EUR = 1). The fitness values obtained for all scenarios are given in Table 9. As can be seen, as the effect of price increases, the cost-effective ZnO nanofluid stands out. The fitness value of ZnO at high temperatures shows very little difference compared to the lowest temperature in cases where the cost is 40% and above (EUR ≥ 0.4).

**Figure 10:**
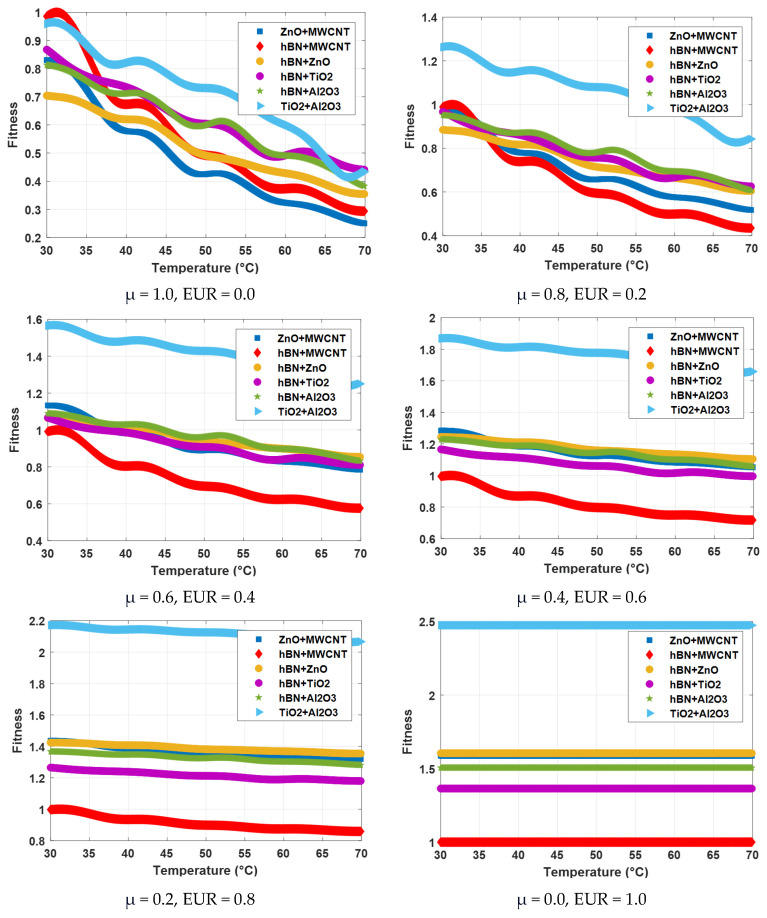
Fitness function results obtained using viscosity and price for hybrid fluids.

Figure 10 shows the fitness values of hybrid nanofluids calculated depending on the temperature increase according to different viscosity weights (μ) and different price weights (EUR). In the case where the viscosity is 100% effective (μ = 1, EUR = 0), hBN + MWCNT has the highest fitness value at the beginning, while the TiO_2_ + Al_2_O_3_ hybrid mixture gives the highest fitness values at other temperatures. In all other scenarios, the TiO_2_ + Al_2_O_3_ hybrid mixture gives the highest fitness values. As can be seen from the graphs, it is understood that the TiO_2_ + Al_2_O_3_ hybrid mixture is the best choice after the cost is added. The fitness values obtained from the hybrid mixtures for all scenarios are given in Table 10. As can be seen, it is clear that the TiO_2_ + Al_2_O_3_ hybrid mixture will be the ideal choice after the effect of price emerges.

**Figure 11:**
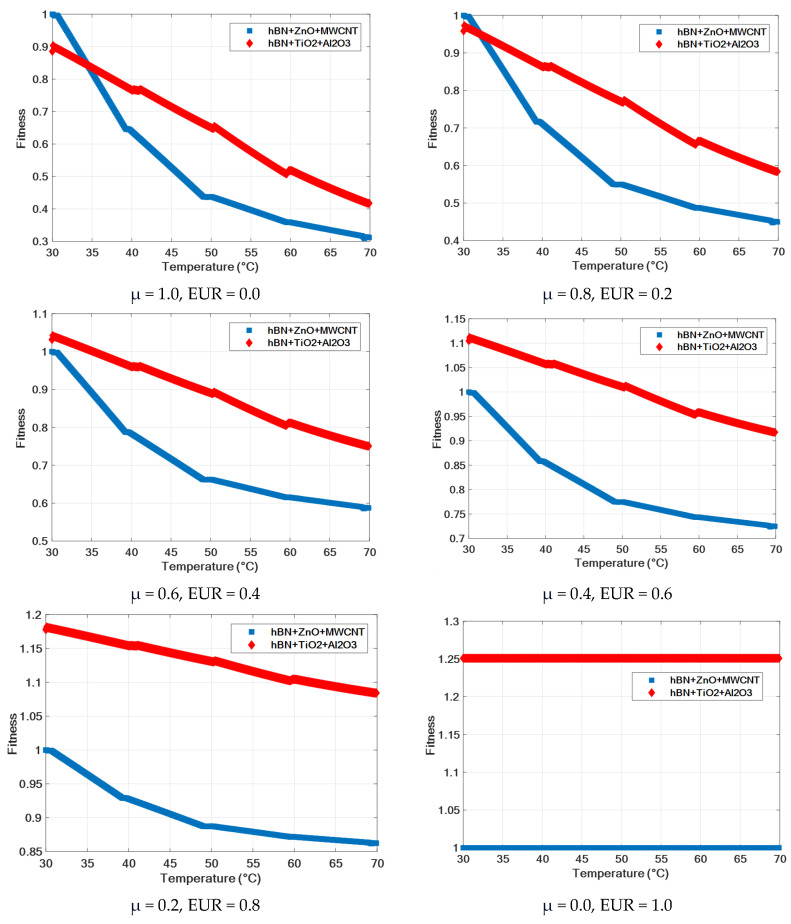
Fitness function results obtained using viscosity and price parameters of ternary fluids.

Figure 11 shows the graphs of the fitness values of ternary nanofluids calculated depending on the temperature increase according to different viscosity weights (μ) and different price weights (EUR). In the case where the viscosity is 100% (μ = 1, EUR = 0), the hBN + ZnO + MWCNT ternary mixture has the highest fitness value at the beginning, while the hBN + TiO_2_ + Al_2_O_3_ ternary mixture gives the highest fitness values at other temperatures. The same applies to the case where the viscosity is 80% (μ = 0.8, EUR = 0.2). In all other scenarios, the hBN + TiO_2_ + Al_2_O_3_ ternary mixture gives the highest fitness values at all temperatures. As can be seen from the graphs, it is understood that the hBN + TiO_2_ + Al_2_O_3_ ternary mixture is the best choice in all scenarios where the cost is 40% and above (EUR ≥ 0.4). Table 11 shows the fitness values obtained from all scenarios for ternary mixtures.

## 4. Conclusions

In this study, the temperature-dependent dynamic viscosity changes in nanofluids and their hybrid and ternary mixtures, which increase efficiency and reduce failures in machining processes, were investigated. Since it is difficult to achieve laboratory conditions for dynamic viscosity measurement at high temperatures, only 30 measurement values (a total of 390 measurements) could be obtained for each nanofluid and mixture (hybrid, ternary). Despite this limitation, reliable predictions were obtained with the proposed GPR method. Acceptable RMSE values (0.24931; 0.34338; 0.41845) were obtained under strong consistency of R^2^ = 1 for single nanofluids, hybrid and ternary mixtures, respectively. Training times were negligible due to data augmentation. In the evaluations made in terms of dynamic viscosity, TiO_2_ can be preferred as the single nanofluid at high temperatures, while a mixture of hBN and TiO_2_ is recommended for hybrid mixtures, and a mixture of hBN, TiO_2_, and Al_2_O_3_ is recommended for ternary mixtures. There may be some practical difficulties when using nanofluids containing TiO_2_, hBN and Al_2_O_3_. These particles may tend to settle, especially during long-term storage; therefore, surface modification, ultrasonic mixing, or dispersion agents may be required to ensure stability. In addition, such nanoparticles can cause aggregation and clogging in filtration systems; this can reduce equipment life and increase the need for regular maintenance. In terms of health, TiO_2_ is classified as a potential carcinogen in respirable form; hBN and Al_2_O_3_, although generally of lower toxicity, may pose a risk when inhaled at nanoscale. In addition, their use at very high operating speeds is not recommended in practice because of the potential for reaction-forming effects.

When the unit prices of nanofluids and their mixtures are evaluated with a fitness function together with dynamic viscosity, it is seen that ZnO should be preferred in cases where the cost factor increases for single nanofluids. In hybrid mixtures, the selection of the ZnO + MWCNT mixture stands out as a more suitable option with the increase in cost. Similarly, for ternary mixtures, the hBN + TiO_2_ + Al_2_O_3_ mixture is expected to be preferred with the increase in cost. The extent of the cost increase is a subject that needs to be discussed, and the analyses conducted show that even a 20% increase in cost can lead to changes in material selection. In future studies, it is envisaged that multi-objective meta-heuristic methods can be used to model the relationship between dynamic viscosity and cost more effectively by applying algorithms, such as genetic algorithms, particle swarm optimization, and ant colony algorithms, and comparing the performance results.

## Figures and Tables

**Table 1:** Technical specifications of sunflower oil *.

Oil Type	Density (15 °C, g/mL)	Viscosity (40 °C, mm^2^/s)	Flash Point (°C)	Appearance	Additions (%)
Sunflower oil	0.888	34.25	130	Clear, Light yellow	Stabilizer: 0.3 Antifoam: 0.0015

* Information was obtained from the manufacturer.

**Table 2:** Properties of the nanoparticles used *.

Nanoparticle Type	Density (g/cm^3^)	Particle Size (nm)	Purity (%)	Color
Hexagonal boron nitride (hBN)	2.29	65–75	99.8	White
Zinc oxide (ZnO)	5.61	18	99.9	White
Multi-walled carbon nanotube (MWCNT)	2.40	48–78	96.0	Black
Titanium dioxide (TiO_2_)	3.90	10–25	99.5	White
Aluminum oxide (Al_2_O_3_)	3.89	13	99.5	White

* Information was obtained from the manufacturer.

**Table 3:** Formulas are used to calculate masses corresponding to volumetric concentrations.

Nanoparticle Volumetric Additive Rate	Nanofluid Volume	Base Fluid Density	Nanoparticle Density	Total Nanofluid Mass (For Verification)
ϕ (%)	∀_n_ (mL)	ρ_b_ (kg/m^3^)	ρ_p_ (kg/m^3^)	m_nf_ = m_np_ + m_bf_ + m_SDS_ (g)
Nanoparticle Volume	Base Fluid Volume	Nanoparticle Mass	Base Fluid Mass	Mass Contribution Rate
∀ _p_ = ϕ ∀ _n_ (mL)	∀_b_ = ∀_n_ − ∀_p_ (mL)	m_p_ = ρ_p_∀_p_ (g)	m_b_ = ρ_b_∀_b_ (g)	ϕ_w_ = m_p_/(m_p_ + m_b_) (%)

**Table 4:** Temperature and dynamic viscosity values of mono nanofluids.

Measurement No.	Pure Oil		hBN		ZnO		MWCNT		TiO_2_		Al_2_O_3_	
	T (°C)	μ (mPa·s)	T (°C)	μ (mPa·s)	T (°C)	μ (mPa·s)	T (°C)	μ (mPa·s)	T (°C)	μ (mPa·s)	T (°C)	μ (mPa·s)
1	31.20	35.67	29.75	44.87	29.45	44.89	30.80	90.44	30.15	75.71	30.45	67.20
2	31.60	35.64	29.40	44.75	29.60	44.76	30.55	90.51	30.35	75.64	30.35	67.15
3	31.50	35.63	29.25	45.07	29.55	45.02	30.65	90.46	30.15	75.49	30.65	67.32
4	31.20	35.64	29.45	44.92	29.40	44.95	30.35	90.57	30.10	75.62	30.75	67.20
5	31.80	35.68	29.60	44.85	29.55	44.77	30.50	90.19	30.20	75.68	30.60	67.24
6	31.10	35.65	29.55	44.95	29.45	44.96	30.75	90.63	30.25	75.48	30.20	67.28
7	39.90	26.91	40.10	37.66	39.85	32.95	39.50	58.50	40.05	65.90	41.05	60.15
8	40.80	26.99	39.85	36.87	40.15	32.93	39.60	58.36	39.75	65.35	40.95	60.12
9	40.35	26.93	39.60	37.15	39.80	32.76	39.65	58.44	40.00	65.32	41.20	60.05
10	40.20	26.90	39.65	37.15	39.75	32.97	39.10	58.49	39.70	65.75	40.85	60.01
11	40.55	26.95	39.90	37.36	40.05	32.78	39.35	58.41	39.55	65.08	41.55	60.32
12	40.00	26.97	39.70	37.20	39.80	32.87	39.20	58.37	39.75	65.29	41.60	60.09
13	49.90	19.88	49.70	29.13	49.85	24.84	49.95	51.60	49.95	58.81	49.90	49.85
14	50.00	19.90	49.85	29.62	50.20	25.51	49.85	51.46	50.45	58.45	50.25	49.60
15	49.70	19.87	49.80	29.49	50.05	25.52	50.45	51.47	50.10	58.83	50.00	48.95
16	50.35	19.85	49.35	29.56	49.55	25.29	50.65	51.60	50.25	58.49	50.20	49.69
17	50.10	19.90	49.50	29.38	50.20	25.31	49.85	51.47	50.30	58.48	50.10	49.66
18	49.95	19.88	49.40	29.25	50.15	25.28	50.45	51.48	50.15	58.71	50.15	49.55
19	60.15	15.66	60.05	24.21	60.55	19.87	60.85	42.14	60.40	47.29	60.40	38.64
20	60.05	15.58	59.65	24.18	59.90	20.06	60.25	42.01	59.95	47.09	59.95	38.75
21	58.90	15.72	59.40	24.19	59.85	19.89	60.10	42.07	60.75	48.01	60.00	38.61
22	59.55	15.55	59.65	24.25	60.15	20.05	60.40	41.98	60.65	47.15	59.20	38.28
23	60.55	15.75	59.70	24.12	60.35	20.01	60.45	42.16	60.55	47.21	58.95	38.54
24	60.20	15.64	59.75	24.18	60.40	19.70	60.35	42.04	60.70	47.05	59.70	39.01
25	69.90	12.79	69.95	20.19	69.55	16.44	70.05	32.48	70.15	36.82	70.50	30.47
26	69.80	12.80	70.25	20.53	70.20	16.35	70.15	32.43	70.15	36.94	70.30	31.10
27	70.05	12.77	71.05	20.45	70.05	16.47	69.55	32.50	70.30	36.74	70.45	30.23
28	69.65	12.85	71.10	20.39	69.95	16.45	69.65	32.36	69.90	36.59	69.95	30.21
29	70.00	12.65	70.55	20.33	70.05	16.39	69.75	32.39	70.35	37.25	70.65	30.36
30	69.40	12.90	70.70	20.45	69.60	16.41	69.65	32.67	70.35	36.49	70.55	30.35

**Table 5:** Temperature and dynamic viscosity values of hybrid nanofluids.

Measurement No.	ZnO + MWCNT		hBN + MWCNT		hBN + ZnO		hBN + TiO_2_		hBN + Al_2_O_3_		TiO_2_ + Al_2_O_3_	
	T (°C)	μ (mPa·s)	T (°C)	μ (mPa·s)	T (°C)	μ (mPa·s)	T (°C)	μ (mPa·s)	T (°C)	μ (mPa·s)	T (°C)	μ (mPa·s)
1	30.75	58.44	31.10	70.48	30.15	49.56	29.95	61.40	29.75	57.41	30.25	67.70
2	30.95	58.37	30.75	70.38	29.80	49.78	29.45	62.03	29.45	57.36	30.55	68.14
3	31.10	58.52	30.65	70.40	29.35	49.83	29.65	61.85	29.25	57.49	30.10	67.66
4	30.85	58.25	30.75	69.98	29.65	49.75	30.05	61.21	29.75	57.09	30.15	67.19
5	30.75	58.62	31.10	70.73	29.95	49.69	29.90	61.09	29.80	57.56	30.05	67.84
6	31.00	58.42	31.05	70.54	29.90	49.72	29.80	61.25	29.60	57.56	30.10	67.90
7	40.80	40.69	41.05	47.58	39.85	43.68	39.85	51.84	40.05	50.14	39.80	57.68
8	40.60	40.82	40.45	47.53	39.90	43.68	40.35	50.98	39.75	49.75	40.05	58.01
9	39.95	40.60	40.55	47.62	40.20	43.78	40.55	51.68	39.80	49.79	40.10	58.13
10	39.85	41.03	40.60	47.65	40.55	43.74	40.20	51.65	39.60	50.03	39.55	57.54
11	40.20	40.46	40.80	47.82	39.95	43.75	40.05	51.95	40.00	50.64	39.40	57.55
12	40.40	40.61	40.15	47.25	40.15	43.65	40.20	51.75	39.60	50.45	39.90	57.15
13	49.95	30.07	50.35	34.76	50.35	34.11	50.40	42.56	50.50	42.55	50.60	51.60
14	50.45	29.94	50.50	34.50	50.25	34.91	50.85	42.58	50.30	41.95	50.35	52.03
15	50.55	30.00	49.95	34.62	49.80	34.78	50.65	42.57	50.35	42.45	50.25	51.45
16	49.85	29.61	50.15	34.65	49.95	34.60	50.20	42.26	50.65	42.95	50.40	51.39
17	50.10	29.98	50.65	34.64	49.90	34.57	50.25	42.61	50.25	42.50	50.55	51.50
18	50.30	30.42	50.20	34.61	50.35	34.64	50.05	42.77	50.35	42.89	50.25	51.30
19	59.65	22.75	59.55	26.31	60.35	30.01	59.75	34.66	60.05	34.61	60.35	42.18
20	60.15	22.81	60.15	26.38	59.95	30.16	60.25	35.21	59.65	35.10	60.05	43.04
21	60.05	22.77	60.00	26.28	60.15	30.17	60.35	35.06	59.75	35.02	60.75	41.98
22	59.95	23.06	59.85	26.35	60.45	30.05	60.15	34.18	59.95	34.15	60.10	42.09
23	60.20	22.64	60.20	26.41	60.55	29.97	59.65	34.40	59.40	34.25	60.10	41.95
24	60.00	22.63	59.65	26.15	60.35	30.29	59.25	34.37	59.40	34.58	60.45	41.85
25	69.75	17.68	69.75	20.65	71.15	25.00	70.10	31.02	70.25	26.69	69.95	30.74
26	69.85	17.52	70.05	20.77	70.45	25.13	69.85	30.96	70.70	25.89	70.25	31.39
27	70.20	17.58	70.20	20.66	69.95	24.88	70.15	30.94	70.95	26.74	70.10	31.01
28	70.45	17.65	69.85	20.75	70.25	25.02	69.95	31.15	70.25	26.89	69.80	30.45
29	70.05	17.81	69.75	20.60	70.65	24.91	70.05	31.20	70.05	26.98	69.85	30.29
30	70.30	17.30	69.80	20.69	70.55	25.07	70.50	31.04	70.80	26.94	70.05	30.85

**Table 6:** Temperature and dynamic viscosity values of ternary nanofluids.

Measurement No.	hBN + ZnO + MWCNT		hBN + TiO_2_ + Al_2_O_3_	
	T (°C)	μ (mPa·s)	T (°C)	μ (mPa·s)
1	30.05	69.48	30.05	62.55
2	30.35	69.02	30.20	62.45
3	30.50	69.20	30.10	62.98
4	30.55	69.28	30.00	60.91
5	30.65	69.25	29.75	62.34
6	30.30	69.14	29.90	62.84
7	39.75	44.77	40.35	53.24
8	39.65	44.95	40.25	53.28
9	39.25	44.82	40.10	53.12
10	39.45	45.11	40.30	53.41
11	39.60	44.67	41.05	53.49
12	39.30	44.75	40.95	53.02
13	50.05	30.41	50.30	45.01
14	49.05	30.28	50.35	44.83
15	49.25	30.36	50.25	44.67
16	49.30	30.56	50.40	45.68
17	49.30	30.24	50.25	45.12
18	49.45	30.25	50.25	45.07
19	59.25	25.06	59.85	35.94
20	59.60	24.87	60.05	36.00
21	59.85	24.96	59.50	35.18
22	59.75	25.09	60.10	35.89
23	59.65	24.80	60.10	36.15
24	59.50	24.94	59.80	36.09
25	69.50	21.84	69.95	29.25
26	69.95	21.66	70.40	30.02
27	69.90	21.68	70.20	28.79
28	69.30	21.93	69.95	28.95
29	69.05	22.03	69.85	28.88
30	69.30	20.93	70.25	29.01

**Table 7:** Performance metrics of regression models.

Material	Parameters	Gaussian Process Regression	Neural Network	SVM	Regression Trees
Mono Nanofluids	RMSE	0.24931	1.8217	1.816	2.0492
	R-squared	1.00	0.99	0.99	0.99
	MSE	0.062157	3.3186	3.2978	4.199
	MAE	0.16108	1.1674	1.7928	1.1412
	Training time	13.113 s	11.449 s	10.395 s	12.23 s
Hybrid Nanofluids	RMSE	0.34338	0.4197	1.3747	2.6424
	R-squared	1.00	1.00	0.99	0.96
	MSE	0.11791	0.17614	1.8897	6.9824
	MAE	0.24463	0.32224	1.3184	1.3477
	Training time	30.524 s	30.621 s	8.9573 s	7.9075 s
Ternary Nanofluids	RMSE	0.4793	0.50341	1.5558	5.1066
	R-squared	1.00	1.00	0.99	0.87
	MSE	0.22972	0.25342	2.4204	26.077
	MAE	0.33597	0.34978	1.4412	3.0038
	Training time	7.7757 s	21.077 s	5.934 s	3.4376 s

**Table 8:** 100 g unit prices of mono, hybrid, and ternary nanofluids used in the study.

Mono	Cost (EUR)	Hybrid	Cost (EUR)	Ternary	Cost (EUR)
hBN	290	ZnO + MWCNT	184	hBN + ZnO + MWCNT	219.3
ZnO	74	hBN + MWCNT	292	hBN + TiO_2_ + Al_2_O_3_	175.3
MWCNT	294	hBN + ZnO	182		
TiO_2_	138	hBN + TiO_2_	214		
Al_2_O_3_	98	hBN + Al_2_O_3_	194		
		TiO_2_ + Al_2_O_3_	118		

**Table 9:** Results obtained using temperature, viscosity, and price parameters of single nanofluids.

Mono Nanofluid	T (°C)	μ (mPa·s)	Fitness					
			μ = 1.0 EUR = 0.0	μ = 0.8 EUR = 0.2	μ = 0.6 EUR = 0.4	μ = 0.4 EUR = 0.6	μ = 0.2 EUR = 0.8	μ = 0.0 EUR = 1.0
hBN	30	44.70	0.49	0.60	0.70	0.81	0.91	1.01
	40	37.36	0.41	0.53	0.65	0.77	0.89	1.01
	50	29.42	0.33	0.46	0.60	0.74	0.88	1.01
	60	24.17	0.27	0.42	0.57	0.72	0.86	1.01
	70	20.31	0.22	0.38	0.54	0.70	0.86	1.01
ZnO	30	44.52	0.49	1.19	1.88	2.58	3.28	3.97
	40	32.86	0.36	1.09	1.81	2.53	3.25	3.97
	50	25.28	0.28	1.02	1.76	2.5	3.23	3.97
	60	19.98	0.22	0.97	1.72	2.47	3.22	3.97
	70	16.41	0.18	0.94	1.7	2.46	3.21	3.97
MWCNT	30	90.30	1	1	1	1	1	1
	40	58.33	0.64	0.72	0.79	0.86	0.93	1
	50	51.52	0.57	0.66	0.74	0.83	0.91	1
	60	42.06	0.46	0.57	0.68	0.79	0.89	1
	70	32.45	0.36	0.49	0.62	0.74	0.87	1
TiO_2_	30	75.63	0.84	1.09	1.35	1.61	1.87	2.13
	40	65.59	0.72	1.01	1.29	1.57	1.85	2.13
	50	58.84	0.65	0.95	1.24	1.54	1.83	2.13
	60	47.10	0.52	0.84	1.16	1.49	1.81	2.13
	70	36.73	0.41	0.75	1.1	1.44	1.79	2.13
Al_2_O_3_	30	67.19	0.74	1.19	1.65	2.1	2.55	3
	40	59.88	0.66	1.13	1.6	2.06	2.53	3
	50	49.59	0.55	1.04	1.53	2.02	2.51	3
	60	38.70	0.43	0.94	1.46	1.97	2.49	3
	70	30.48	0.34	0.87	1.4	1.93	2.47	3

**Table 10:** Results obtained using temperature, viscosity, and price for hybrid fluids.

Hybrid Nanofluid	T (°C)	μ (mPa·s)	Fitness					
			μ = 1.0 EUR = 0.0	μ = 0.8 EUR = 0.2	μ = 0.6 EUR = 0.4	μ = 0.4 EUR = 0.6	μ = 0.2 EUR = 0.8	μ = 0.0 EUR = 1.0
ZnO + MWCNT	30	58.57	0.83	0.98	1.13	1.28	1.44	1.59
	40	40.76	0.58	0.78	0.98	1.18	1.39	1.59
	50	29.96	0.42	0.66	0.89	1.12	1.35	1.59
	60	22.78	0.32	0.58	0.83	1.08	1.33	1.59
	70	17.62	0.25	0.52	0.78	1.05	1.32	1.59
hBN + MWCNT	30	69.54	0.99	0.99	0.99	0.99	1	1
	40	47.48	0.67	0.74	0.80	0.87	0.93	1
	50	34.7	0.49	0.59	0.70	0.80	0.90	1
	60	26.32	0.37	0.50	0.62	0.75	0.87	1
	70	20.68	0.29	0.43	0.58	0.72	0.86	1
hBN + ZnO	30	49.66	0.70	0.88	1.06	1.24	1.42	1.60
	40	43.72	0.62	0.82	1.01	1.21	1.41	1.60
	50	34.64	0.49	0.71	0.94	1.16	1.38	1.60
	60	30.2	0.43	0.66	0.90	1.13	1.37	1.60
	70	24.99	0.35	0.60	0.85	1.10	1.35	1.60
hBN + TiO_2_	30	61.19	0.87	0.97	1.07	1.17	1.27	1.36
	40	51.78	0.73	0.86	0.99	1.11	1.24	1.36
	50	42.59	0.60	0.76	0.91	1.06	1.21	1.36
	60	34.68	0.49	0.67	0.84	1.02	1.19	1.36
	70	31.07	0.44	0.63	0.81	0.99	1.18	1.36
hBN + Al_2_O_3_	30	57.32	0.81	0.95	1.09	1.23	1.37	1.51
	40	50.17	0.71	0.87	1.03	1.19	1.35	1.51
	50	42.29	0.60	0.78	0.96	1.14	1.32	1.51
	60	34.59	0.49	0.69	0.90	1.10	1.30	1.51
	70	26.97	0.38	0.61	0.83	1.06	1.28	1.51
TiO_2_ + Al_2_O_3_	30	67.6	0.96	1.26	1.57	1.87	2.17	2.47
	40	57.77	0.82	1.15	1.48	1.81	2.14	2.47
	50	51.57	0.73	1.08	1.43	1.78	2.13	2.47
	60	42.44	0.60	0.98	1.35	1.73	2.10	2.47
	70	30.62	0.43	0.84	1.25	1.66	2.07	2.47

**Table 11:** Results obtained using temperature, viscosity, and price parameters of ternary fluids.

Ternary Nanofluid	T (°C)	μ (mPa·s)	Fitness					
			μ = 1.0 EUR = 0.0	μ = 0.8 EUR = 0.2	μ = 0.6 EUR = 0.4	μ = 0.4 EUR = 0.6	μ = 0.2 EUR = 0.8	μ = 0.0 EUR = 1.0
hBN + ZnO + MWCNT	30	69.46	1	1	1	1	1	1
	40	44.38	0.64	0.71	0.78	0.86	0.93	1
	50	30.38	0.44	0.55	0.66	0.77	0.89	1
	60	24.92	0.36	0.49	0.62	0.74	0.87	1
	70	21.68	0.31	0.45	0.59	0.72	0.86	1
hBN + TiO_2_ + Al_2_O_3_	30	61.51	0.89	0.96	1.03	1.10	1.18	1.25
	40	53.25	0.77	0.86	0.96	1.06	1.15	1.25
	50	45.18	0.65	0.77	0.89	1.01	1.13	1.25
	60	35.99	0.52	0.66	0.81	0.96	1.10	1.25
	70	29.00	0.42	0.58	0.75	0.92	1.08	1.25

## Data Availability

The original contributions presented in this study are included in the article. Further inquiries can be directed to the corresponding author.
